# Comorbidity in Adult Bone Sarcoma Patients: A Population-Based Cohort Study

**DOI:** 10.1155/2014/690316

**Published:** 2014-02-27

**Authors:** Ninna Aggerholm-Pedersen, Katja Maretty-Nielsen, Johnny Keller, Steen Baerentzen, Akmal Safwat

**Affiliations:** ^1^Sarcoma Centre of Aarhus University Hospital, Norrebrogade 44, 8000 Aarhus, Denmark; ^2^Department of Experimental Clinical Oncology, Aarhus University Hospital, Norrebrogade 44, Building 5, 8000 Aarhus, Denmark; ^3^Department of Oncology, Aarhus University Hospital, Norrebrogade 44, Building 5, 8000 Aarhus, Denmark; ^4^Department of Orthopedic Surgery E5, Aarhus University Hospital, Norrebrogade 44, Building 7, 8000 Aarhus, Denmark; ^5^Department of Pathology, Aarhus University Hospital, Norrebrogade 44, Building 18, 8000 Aarhus, Denmark

## Abstract

*Background*. Comorbidity is an important prognostic factor for survival in different cancers; however, neither the prevalence nor the impact of comorbidity has been investigated in bone sarcoma. *Methods*. All adult bone sarcoma patients from western Denmark treated at the Aarhus Sarcoma Centre in the period from 1979 to 2008 were identified through a validated population-based database. Charlson Comorbidity Index scores were computed, using discharge diagnoses from the Danish National Patient Registry. Survival was assessed as overall and disease-specific mortality. The impact of comorbidity was examined as rates according to the level of comorbidity as well as uni- and multivariately using proportional hazard models. *Results*. A total of 453 patients were identified. The overall prevalence of comorbidity was 19%. The prevalence increased with age and over the study period. In patients with Ewing/osteosarcoma, comorbidity was not associated with an increased overall or disease-specific mortality. However, patients with bone sarcomas other than Ewing/osteosarcoma had increased overall mortality. Independent prognostic factors for disease-specific survival were age, tumor size, stage at diagnosis, soft tissue involvement, grade, and surgery. *Conclusion*. The prevalence of comorbidity in bone sarcoma patients is low. Comorbidity impaired survival in patients with non-Ewing/nonosteosarcoma, histology. This emphasizes the importance of not only treating the sarcoma but also comorbidity.

## 1. Introduction

Bone sarcoma is a rare disease, with an incidence of approximately 8 cases per million/year. It occurs in all ages but has a characteristic bimodal distribution, with peak incidences for adolescents and elderly [1]. Changes in the general population are expected in the future, resulting in an increased population of elderly. The treatment of these is often complicated by the presence of chronic diseases, for example, comorbidity, which may impact survival. Comorbidity is an important prognostic factor for survival in other cancers, such as head and neck, renal, and bladder cancer [2–7]. Several factors have previously been identified as prognostic in bone sarcoma patients; however the impact of comorbidity has not been investigated previously.

The structure of the Danish health care system, with free of charge health care for all residents, and the extensive use of population-based health registries provide a unique possibility to examine the impact of comorbidity in large population-based series. We have just published an article covering the impact of comorbidity on overall survival in soft tissue sarcoma patients treated in the same institute over the same period of time. Because of the differences in age distribution, prognosis, pathological types, treatment modalities, and outcome between adult soft tissue and bone sarcomas, we chose to report the results in two separate publications. The aim of this study was therefore to examine the prevalence of comorbidity and its impact on survival among adult bone sarcoma patients.

## 2. Patients and Methods

The population of western Denmark is approximately 2.5 million [8]. All Danish residents are assigned a unique 10-digit civil personal registration number (CPR number), rendering individual linkage throughout all Danish registries possible [9, 10].

### 2.1. Identification of Bone Sarcoma Patients

Since 1979 all sarcoma patients treated at the Aarhus Sarcoma Centre have been registered in a population-based clinical database, the Aarhus Sarcoma Registry [1]. Patients at the Aarhus Sarcoma Centre are diagnosed and treated, according to national guidelines, by an experienced multidisciplinary sarcoma team [11, 12]. Bone sarcomas were classified primarily as low or high grade, with the exception of chondrosarcoma being classified as low, intermediate, or high grade.

**Figure 1:**
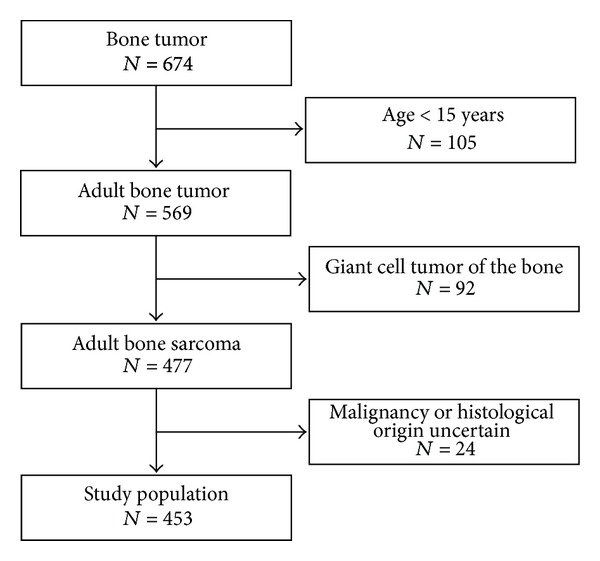
Flow chart for patients diagnosed with a bone sarcoma at the Sarcoma Centre of Aarhus University Hospital in the period from 1979 to 2008.

Between 1979 and 2008, 651 consecutive patients were treated for bone sarcomas or related lesions at the Sarcoma Centre of Aarhus University Hospital, Denmark. Patients were excluded as shown in Figure 1. The study population comprised 453 adult patients.

### 2.2. Comorbidity

The National Patient Registry (NPR) contains information on all somatic patients admitted to Danish hospitals since 1977, as well as outpatient visits since 1995 [13–16]. Registered data includes CPR number, admission and discharge dates, as well as all discharge diagnoses according to the eighth (prior to 1994) or tenth version of the International Classification of Disease (ICD-8 and ICD-10). The registry covers more than 99% of all Danish hospital admissions [16].

Comorbidity was assessed using the Charlson Comorbidity Index [17]. The Charlson Comorbidity Index was originally developed to predict 1-year mortality in a cohort of 559 medical patients and has later been adapted for usage with ICD-based hospital discharge data [18]. The index includes 19 medical conditions, which are weighted from 1 to 6 (Table 1) according to the risk of mortality and added to form a final score [17]. The included ICD codes are shown in Supplementary Materials (see Supplementary Table 1 in Supplementary Materials available online at http://dx.doi.org/10.1155/2014/690316).

The 453 adult bone sarcoma patients in the ASR were linked through their CPR number to the NPR and all discharge diagnoses registered before the date of the sarcoma diagnosis were extracted. Based on these diagnoses, a Charlson Comorbidity score for each patient was computed. All discharge diagnoses of 30 days and all cancer diagnoses of 90 days prior to the sarcoma diagnosis were excluded to eliminate diagnoses related to the sarcoma.

### 2.3. Survival

Survival was assessed using overall and disease-specific mortality as endpoints. Patients were followed from the date of sarcoma diagnosis until death, emigration, or end of the study period. Data on death was obtained through the Danish Civil Registration system, which was established in 1968 and comprises current and historical information on Danish residents. Data includes CPR number, date of birth, residence, vital status (dead/alive), and date of death. The vital status is updated on a daily basis [9, 10].

The cause of death was obtained from the ASR and the Danish Cause of Death Registry [19]. The Danish Cause of Death Registry was initiated in 1875 based on the mandatory completion of death certificates for any death occurring in Denmark. The registry contains medical information from the death certificates including the immediate and underlying cause of death according to the ICD-8 and ICD-10 [19]. Disease-specific mortality was defined as death from sarcoma (ICD-8; 170, 171, 192.49–99 and ICD-10; C40-C41, C47, C49) or death with known metastatic disease.

### 2.4. Statistical Analyses

Comorbidity was analyzed as a categorical value based on the Charlson Comorbidity score as follows: no (score 0), mild (score 1), moderate (score 2), and severe (score ≥ 3) comorbidities.The prevalence and type of comorbidity were assessed as proportions. Patient characteristics according to the level of comorbidity were analyzed using the Mann-Whitney *U* test and the chi-square test. Overall and disease-specific mortality was assessed as rates with 95% confidence intervals (CI) and presented as cumulative incidence functions according to the level of comorbidity [20–23]. The association between comorbidity and mortality was assessed both uni- and multivariately, adjusting for the following possible prognostic factors: age, stage at diagnosis, tumor size, soft tissue involvement, grade, histological type, surgery, and chemotherapy. These were included as seen in Table 2. The correlation between the continuous variables (age and tumor size) and mortality was examined using the likelihood ratio test, comparing models with inclusion of the variables as continuous linear and as four-knotted cubic splines, respectively. No significant difference between the respective models was found (overall mortality: age *P* = 0.11, tumor size *P* = 0.22; disease specific mortality: age *P* = 0.73, tumor size *P* = 0.13), and age and tumor size were thus included as continuously linear variables [24–26]. Missing data on tumor size and margin were computed using multiple imputations by chained equations [27]. Crude and adjusted hazard ratios with 95% CI were computed using the Cox proportional hazard model. The proportional hazard assumption was assessed graphically. Disease-specific mortality was analyzed with death from other causes as a competing risk [28]. Effect modification was tested using the likelihood ratio test and assessed according to the principles described by Oxman and Guyatt [29]. A significant interaction between comorbidity and the histological subtype was encountered (*P* = 0.0003) and stratum-specific hazard ratios were thus computed. All tests were two-sided and a *P* value ≤ 0.05 was considered significant. Analyses were employed using Stata, version 13.0.

### 2.5. Ethics

This study was approved by the Danish Data Protection Agency and the Danish Health and Medicines Authority.

## 3. Results

### 3.1. Patient Characteristics

Overall, 453 adult patients were diagnosed with a bone sarcoma in western Denmark between 1979 and 2008. The median age was 46 years (range 15–90) and 60% were males. The patient characteristics according to the Charlson Comorbidity Index score are shown in Table 3. The level of comorbidity was significantly associated with increased age, diagnosis in the last part of the study period, no surgery, a higher proportion of amputations, intralesional/marginal excisions, chemotherapy, and palliative treatment. As seen in Table 4, the most frequent histological types were chondrosarcoma and osteosarcoma. The median followup was 5.9 years (range 0.0–34.1).

### 3.2. Prevalence of Comorbidity

**Figure 2:**
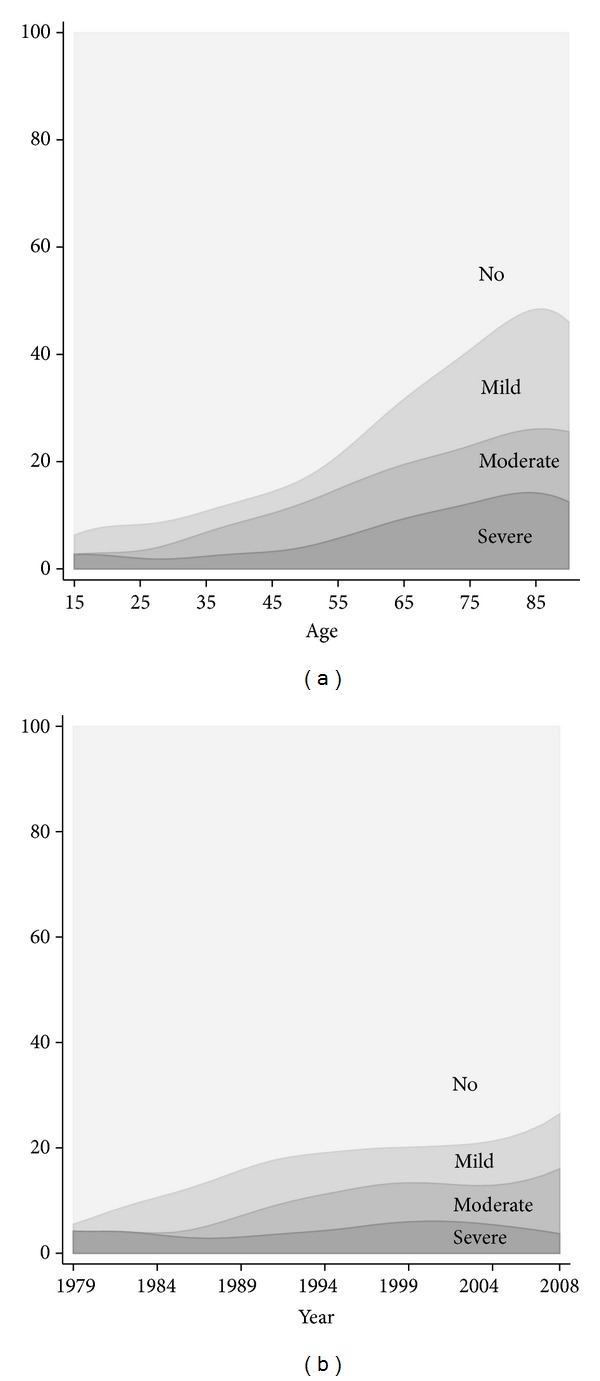
The prevalence of comorbidity as a percentage by age (a) and calendar year of diagnosis (b) in adult bone sarcoma patients treated at the Sarcoma Centre of Aarhus University Hospital in the period from 1979 to 2008 (*N* = 453).

Comorbidity was present in 85 of the 453 adult bone sarcoma patients, corresponding to a prevalence of 19%. The prevalence of comorbidity was 15% and 21% for patients with Ewing/osteosarcoma and non-Ewing/nonosteosarcoma, respectively. Mild comorbidity was seen in 44% of the patients with comorbidity, while moderate and severe comorbidities were seen in 32% and 25%, respectively. As seen in Table 1, the most frequent type of comorbidity was “any tumor,” which was seen in 5.5% of the patients. The prevalence of overall comorbidity increased with increasing age, being most frequent at 86 years where 44% of the patients had comorbidity (Figure 2(a)). As seen in Figure 2(b) the prevalence of comorbidity increased over the study period, from 6% in 1979 to 26% in 2008. The prevalence of severe comorbidity was nearly constant, while mild and moderate comorbidities increased over time from 1% to 10% and from 0% to 12%, respectively.

### 3.3. Overall Mortality

**Figure 3:**
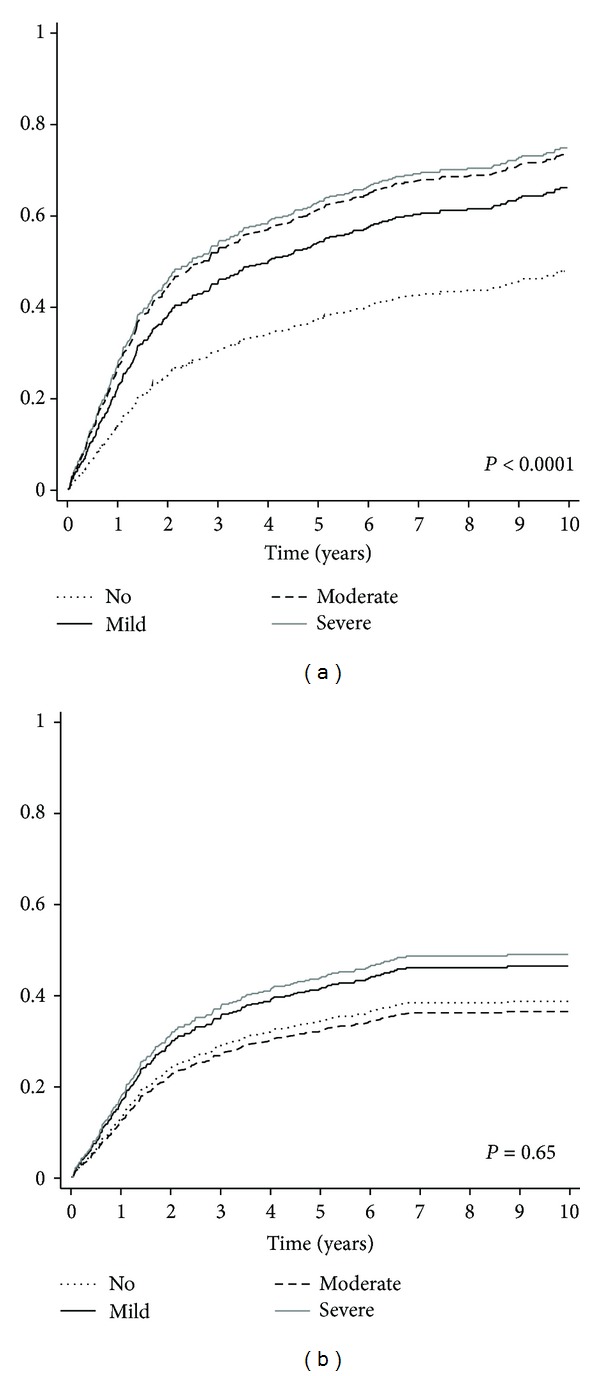
The crude cumulative incidence function of overall (a) and disease-specific mortality (b) by Charlson Comorbidity score.

In total, the 5- and 10-year overall mortality was 41% (95% CI 37–46) and 52% (95% CI 47–57), respectively. The crude overall mortality was significantly higher in patients with comorbidity, independent of the level, as shown in Figure 3(a) and Table 2. Increasing age and tumor size, metastases at diagnosis, soft tissue involvement, high grade, and intralesional/marginal excision or no surgery was independently significantly associated with an increased overall mortality (Table 2). Moderate and severe comorbidities were found to be independent, significant prognostic factors for overall mortality in patients with non-Ewing/nonosteosarcoma histology. Comorbidity was not associated with increased mortality in Ewing/osteosarcoma patients (Table 5).

### 3.4. Disease-Specific Mortality

In total 188 of the 453 patients died of their bone sarcoma or with metastatic disease, corresponding to a 5- and 10-year disease-specific mortality of 36% (95% CI 31–40) and 40% (95% CI 36–45), respectively. The cumulative incidence function of the crude disease-specific mortality by level of comorbidity is shown in Figure 3(b). For patients without comorbidity, the crude 5-year disease-specific mortality was 34% (95% CI 30–39), while for patients with mild, moderate, and severe comorbidities it was 46% (95% CI 30–62), 37% (95% CI 19–55), and 43% (95% CI 22–64), respectively. The level of comorbidity was not significantly correlated with disease-specific mortality in neither the uni- nor multivariate analysis as shown in Tables 2 and 5. Independent adverse prognostic factors were increasing age and tumor size, metastasis at diagnosis, soft tissue involvement, high grade, and intralesional/marginal excision or no surgery.

## 4. Discussion

In this population-based study of 453 adult bone sarcoma patients we found an overall prevalence of comorbidity of 19%. The prevalence of comorbidity increased with an increasing age and over the study period. Independent adverse prognostic factors for disease-specific survival were increasing age and tumor size, metastasis at diagnosis, soft tissue involvement, high grade, and intralesional/marginal excision or no surgery. Moderate and severe comorbidities were significantly associated with overall survival in patients with non-Ewing/nonosteosarcoma histology, even when adjusting for important prognostic factors.

### 4.1. Methodological Reflections

The main strength of our study is the large sample size as well as population-based data with complete followup on all patients, facilitated by the structure of the Danish health care system. The information on comorbidity was extracted from an administrative registry. The potential information bias is considered low, especially compared to studies based on self-administered questionnaires or medical files, where comorbidity is suspected to be underreported. Coding errors in an administrative registry are expected to some extent; however since the comorbidity occurred before the sarcoma diagnosis, any misclassification is expected to be nondifferential [30]. One of the limitations of this study is the fact that outpatient data was only registered after 1995, indicating that minor comorbidity not requiring hospital admission is only captured in the last half of the study period. Furthermore the NPR was initiated in 1977, leaving only two years of registered information on comorbidity for patients diagnosed with bone sarcoma in 1979. Yet, the increase in prevalence was uniform throughout the entire study period suggesting that comorbidity missed on this basis is minor.

The use of the ICD-10 codes in the NPR for the medical conditions in the Charlson Comorbidity Index has previously been validated. Thygesen et al. [31] reported an overall positive predictive value of 98% for the 19 conditions. The lowest positive predictive value was seen for diabetes mellitus with end organ damage (82%). The prevalence of some of the milder conditions is expected to be underestimated in the NPR, since low-prevalent, severe diseases generally tend to have high negative predictive value, while high-prevalent, mild diseases tend to have lower negative predictive values. However the negative predictive value for ICD coding in the NPR has, to our knowledge, not been investigated.

The Charlson Comorbidity Index has previously been validated for various cancer types [3–6, 18, 32, 33]. The index does not perfectly adjust for comorbidity since some of the medical conditions with the same weight have different outcomes, for example, myocardial infarction and connective tissue disease. Furthermore the prevalence and prognosis for some of the 19 medical conditions have changed radically since the origin, and an update of the index is relevant. Other comorbidity indices exist; however the results from most of these are comparable [7, 34, 35].

Survival was assessed as overall and disease-specific mortality. Disease-specific mortality relies on precise and correct data on the cause of death, and particularly in elderly patients where comorbidity is common it can be difficult to achieve reliable data. Furthermore in patients with a preceding cancer diagnosis, the risk of stating the cancer as cause of death is increased, causing differential misclassification. Data for the cause of death was retrieved from the ASR in the majority of the cases, where the information is expected to be more precise than the Registry of Cause of Death.

### 4.2. Prevalence

The prevalence of comorbidity was 19%. The prevalence in our study was low, as expected, since the prevalence of comorbidity increases with increasing age and the median age in our study was only 46 years. The prevalence of comorbidity in bone sarcoma has not previously been investigated, even though a study of 27,506 primary cancer patients (including 413 musculoskeletal tumors) reported comorbidity in 65% of the overall cases. This study did however include more conditions than the Charlson Comorbidity Index and the musculoskeletal tumor represented only a minor proportion [36]. Comorbidity has been reported to be prevalent in 30–40% of cases in other cancer types; however, the median age in these types is considerably higher [3–6, 32]. A study of melanoma patients, where 47% of the patients were younger than 55 years, reported a prevalence of 19% [37]. We found that the overall prevalence increased over the study period, consistently with the existing literature [4–6, 32].

### 4.3. Survival

The overall and disease-specific mortality rates reported were comparable to the findings of other studies [38, 39]. The impact of comorbidity was significantly different in patients with Ewing/osteosarcoma histology compared to patients with other subtypes. A tendency towards comorbidity being associated with increased overall and disease-specific mortality was observed in patients with non-Ewing/nonosteosarcoma histology. Comorbidity was not associated with neither overall nor disease-specific survival in Ewing/osteosarcoma patients. This might be explained by the low number of patients with comorbidity in this group and thus the low power. Ewing/osteosarcoma patients with severe comorbidity had a surprisingly low overall and disease-specific mortality. When reviewing these eight patients, three were previously cured from cancer with a good prognosis, and one was primarily diagnosed with an unspecified tumor, later reviewed as Ewing sarcoma. All of these patients are still alive, which might contribute to the low mortality rate.

The impact of comorbidity on survival in bone sarcoma has not, to our knowledge, been investigated. Comorbidity has been found to significantly impact survival in various other cancers [2–6, 32].

## 5. Conclusion

The prevalence of comorbidity in adult bone sarcoma patients is low. The level of comorbidity seemingly did not impact the level of treatment in patients with Ewing/osteosarcoma and thus not the disease-specific mortality. Moderate and severe comorbidities were significantly associated with overall survival in patients with non-Ewing/nonosteosarcoma histology, even when adjusting for important prognostic factors. This emphasizes the importance of not only treating the sarcoma but also the comorbidity.

## Supplementary Material

The supplementary table, shows the ICD-8 and ICD-10 codes used to calculate the Charlson Comorbidity Index used in this analysis.

## Figures and Tables

**Table 1:** Prevalence and scores of medical conditions as listed in the Charlson Comorbidity Index among adult bone sarcoma patients treated at the Aarhus Sarcoma Centre between 1979 and 2008 (*N* = 453).

Conditions	*N*	%	Score
Myocardial infarct	9	2.0	1
Congestive heart failure	5	1.1	1
Peripheral vascular disease	12	2.7	1
Cerebrovascular disease	9	2.0	1
Dementia	0	0.0	1
Chronic pulmonary disease	15	3.3	1
Connective tissue disease	6	1.3	1
Ulcer disease	10	2.2	1
Mild liver disease	1	0.2	1
Diabetes	11	2.4	1
Hemiplegia	0	0.0	2
Moderate/severe renal disease	2	0.4	2
Diabetes with end organ damage	1	0.2	2
Any tumor^a^	25	5.5	2
Leukemia	3	0.7	2
Lymphoma	3	0.7	2
Moderate/severe liver disease	0	0.0	3
Metastatic solid tumor	8	1.8	6
AIDS	0	0.0	6

^a^Excluding tumors in soft tissue and bone (ICD-8; 170, 171, 192.49–99 and ICD-10; C40-C41, C47, C49).

**Table 2:** Uni- and multivariate analyses of comorbidity and possible important prognostic factors for overall and disease-specific mortality in adult bone sarcoma patients (*N* = 453).

	*N* (%)	Overall mortality			Disease-specific mortality		
		5-year (%)	HR (95% CI)		5-year (%)	HR (95% CI)	
			Univariate	Multivariate*		Univariate	Multivariate*
Age							
1 year	—	—	1.03 (1.02-1.03)	1.03 (1.02–1.04)	—	1.01 (1.00–1.02)	1.02 (1.01–1.03)
Comorbidity							
No	368 (81)	38	1	1	34	1	1
Mild	37 (8)	52	1.64 (1.08–2.49)	1.11 (0.71–1.75)	46	1.47 (0.90–2.40)	1.08 (0.63–1.86)
Moderate	27 (6)	52	2.03 (1.26–3.25)	1.64 (0.99–2.72)	37	1.29 (0.68–2.46)	1.20 (0.61–2.36)
Severe	21 (5)	62	2.25 (1.37–3.70)	1.35 (0.80–2.27)	43	1.79 (0.97–3.31)	1.28 (0.68–2.43)
Stage at diagnosis							
Localized	387 (85)	35	1	1	28	1	1
Metastatic	66 (15)	80	3.97 (2.96–5.33)	2.01 (1.35–2.98)	79	5.55 (4.03–7.64)	2.12 (1.39–3.24)
Tumor size							
1 cm	—	—	1.07 (1.04–1.09)	1.04 (1.02–1.07)	—	1.07 (1.05–1.10)	1.04 (1.01–1.07)
Soft tissue involvement							
No	86 (19)	16	1	1	12	1	1
Yes	367 (81)	47	2.34 (1.60–3.40)	1.72 (1.16–2.56)	41	3.48 (2.05–5.90)	2.08 (1.20–3.62)
Grade							
1	107 (24)	15	1	1	9	1	1
2	69 (15)	22	1.24 (0.76–2.01)	0.86 (0.52–1.42)	16	1.60 (0.81–3.17)	1.14 (0.57–2.29)
3	277 (61)	56	2.70 (1.92–3.78)	2.38 (1.61–3.51)	51	5.05 (3.06–8.33)	3.57 (2.04–6.23)
Histology							
Ewing/osteosarcoma	176 (39)	55	1	1	53	1	1
Others	277 (61)	33	0.72 (0.56–0.92)	0.78 (0.55–1.11)	25	0.44 (0.33–0.59)	0.67 (0.44–1.01)
Surgery							
Wide/radical	269 (60)	34	1	1	28	1	1
Intralesional/marginal	117 (26)	34	1.29 (0.96–1.74)	1.66 (1.21–2.28)	27	1.23 (0.86–1.77)	1.77 (1.20–2.60)
No	64 (14)	84	5.59 (4.09–7.65)	2.49 (1.66–3.72)	84	7.01 (4.97–9.89)	3.02 (1.94–4.68)
Chemotherapy							
Curative	106 (23)	40	1	1	39	1	1
Palliative	29 (6)	97	6.71 (4.18–10.79)	1.63 (0.93–2.86)	97	5.63 (3.48–9.13)	1.40 (0.78–2.51)
No	318 (70)	37	1.22 (0.89–1.68)	1.16 (0.76–1.76)	29	0.83 (0.59–1.17)	1.21 (0.76–1.91)

Note: abbreviations: HR: hazard ratio, CI: confidence interval. *Multivariate analyses adjusted mutually for age, comorbidity, stage at diagnosis, tumor size, soft tissue involvement, grade, histology type, surgery, and chemotherapy.

**Table 3:** Patient characteristics by Charlson Comorbidity Index score (*N* = 453).

	*N* (%)	Charlson Comorbidity Index score *N* (%)				*P*-value
		0	1	2	3+	
Number	453 (100)	368 (81)	37 (8)	27 (6)	21 (5)	
Age (years)						
Median (range)	46 (15–90)	40 (15–90)	62 (17–83)	59 (17–81)	64 (21–86)	<0.001
Sex						
Female	179 (40)	137 (37)	20 (54)	12 (44)	10 (48)	
Male	274 (60)	231 (63)	17 (46)	15 (56)	11 (52)	0.18
Year of diagnosis						
1979–1988	131 (29)	116 (32)	10 (27)	0 (0)	5 (24)	
1989–1998	129 (28)	103 (28)	8 (22)	12 (44)	6 (29)	
1999–2008	193 (43)	149 (40)	19 (51)	15 (56)	10 (48)	0.027
Stage at diagnosis						
Localized	387 (85)	321 (87)	32 (86)	18 (67)	16 (76)	
Metastatic	66 (15)	47 (13)	5 (14)	9 (33)	5 (24)	0.018
Tumor size (cm)						
Median (range)^a^	8 (1–30)	8 (1–30)	8 (2–30)	7 (2–15)	10 (3–23)	0.14
Soft tissue involvement						
No	86 (19)	71 (19)	5 (14)	4 (15)	6 (29)	
Yes	367 (81)	297 (81)	32 (86)	23 (85)	15 (71)	0.51
Malignancy grade						
1	107 (24)	86 (23)	11 (30)	7 (26)	3 (14)	
2	69 (15)	55 (15)	8 (22)	4 (15)	2 (10)	
3	277 (61)	227 (62)	18 (50)	16 (59)	16 (76)	0.59
Histological type						
Ewing/osteosarcoma	176 (39)	149 (40)	10 (27)	9 (33)	8 (38)	
Others	277 (61)	219 (60)	27 (73)	18 (67)	13 (62)	0.40
Treatment						
Surgery	389 (86)	325 (88)	30 (81)	19 (70)	15 (71)	0.009
Type^b^						
Resection	257 (66)	213 (66)	19 (63)	13 (76)	12 (80)	
Amputation	130 (34)	112 (34)	11 (37)	4 (24)	3 (20)	<0.001
Margin^c^						
Wide/radical	269 (70)	228 (70)	19 (63)	13 (76)	9 (60)	
Intralesional/marginal	117 (30)	96 (30)	11 (37)	4 (24)	6 (40)	<0.001
Radiotherapy	83 (18)	71 (19)	5 (14)	3 (11)	4 (19)	0.63
Chemotherapy	135 (30)	119 (32)	4 (11)	8 (30)	4 (19)	0.005
Curative	106 (79)	98 (82)	2 (50)	3 (38)	3 (75)	
Palliative	29 (21)	21 (18)	2 (50)	5 (63)	1 (25)	0.012

Note: ^a^tumor size: 38 missing. ^b^Type of surgery: 2 missing. ^c^Surgical margin: 3 missing.

**Table 4:** Histological subtypes in adult bone sarcoma patients treated at the Sarcoma Centre of Aarhus University Hospital from 1979 to 2008 (*N* = 453).

Subtype	*N*	(%)
Chondrosarcoma	197	43.5
Osteosarcoma	136	30.0
Ewing sarcoma	40	8.8
Malignant fibrous histiocytoma	33	7.3
Chordoma	21	4.6
Malignant giant cell tumor	9	2.0
Angiosarcoma	7	1.6
Leiomyosarcoma	5	1.1
Others	3	0.7
Unclassifiable	2	0.4

**Table 5:** Multivariate analyses for the effect of comorbidity on overall and disease-specific mortality according to histological subtype.

Histological subtype	No. of patients	Overall mortality		Disease-specific mortality	
		5-year (%)	HR (95% CI)	5-year (%)	HR (95% CI)
Ewing/osteosarcoma					
No	149	54	1	53	1
Mild	10	70	1.02 (0.43–2.42)	60	0.80 (0.31–2.09)
Moderate	9	67	1.12 (0.47–2.66)	56	0.88 (0.33–2.36)
Severe	8	38	0.79 (0.27–2.32)	37	0.67 (0.20–2.26)
Others					
No	219	30	1	22	1
Mild	27	45	1.42 (0.81–2.48)	41	1.54 (0.78–3.05)
Moderate	18	44	2.36 (1.18–4.70)	28	1.05 (0.37–2.97)
Severe	13	77	2.44 (1.30–4.58)	46	2.07 (0.94–4.56)

Note: abbreviations: HR: hazard ratio, CI: confidence interval. Adjusted for age, stage at diagnosis, tumor size, soft tissue involvement, malignancy grade, surgical margin, and chemotherapy.
